# Exploiting structure similarity in refinement: automated NCS and target-structure restraints in *BUSTER*

**DOI:** 10.1107/S0907444911056058

**Published:** 2012-03-16

**Authors:** Oliver S. Smart, Thomas O. Womack, Claus Flensburg, Peter Keller, Włodek Paciorek, Andrew Sharff, Clemens Vonrhein, Gérard Bricogne

**Affiliations:** a Global Phasing Ltd, Sheraton House, Castle Park, Cambridge CB3 0AX, England

**Keywords:** *BUSTER*, NCS restraints, target-structure restraints, local structural similarity restraints

## Abstract

Maximum-likelihood X-ray macromolecular structure refinement in *BUSTER* has been extended with restraints facilitating the exploitation of structural similarity. The similarity can be between two or more chains within the structure being refined, thus favouring NCS, or to a distinct ‘target’ structure that remains fixed during refinement. The local structural similarity restraints (LSSR) approach considers all distances less than 5.5 Å between pairs of atoms in the chain to be restrained. For each, the difference from the distance between the corresponding atoms in the related chain is found. LSSR applies a restraint penalty on each difference. A functional form that reaches a plateau for large differences is used to avoid the restraints distorting parts of the structure that are not similar. Because LSSR are local, there is no need to separate out domains. Some restraint pruning is still necessary, but this has been automated. LSSR have been available to academic users of *BUSTER* since 2009 with the easy-to-use -autoncs and -­target target.pdb options. The use of LSSR is illustrated in the re-refinement of PDB entries 5rnt, where -target enables the correct ligand-binding structure to be found, and 1osg, where -autoncs contributes to the location of an additional copy of the cyclic peptide ligand.

## Abstract 2

Local structural similarity restraints (LSSR) provide a novel method for exploiting NCS or structural similarity to an external target structure. Two examples are given where *BUSTER* re-refinement of PDB entries with LSSR produces marked improvements, enabling further structural features to be modelled.

## 1. Introduction

The refinement of proteins and other macromolecular structures normally requires the use of geometry restraints because at typical resolutions there are not enough X-ray data for them alone to adequately define the position of each atom (Blow, 2002 ▶; Rupp, 2009 ▶). Geometry restraints provide a method for using additional information about the stereochemistry of the molecule being refined. Engh & Huber (1991 ▶, 2001 ▶) showed how information from small-molecule crystal structures could provide high-quality stereochemical restraints that are used in practically all contemporary protein structure determinations. Noncrystallographic symmetry (NCS) arises when there are two or more copies of a protein (or other macromolecule) within the asymmetric unit of the crystal. These copies generally have similar but not identical structures (Kleywegt, 1996 ▶). Correctly using NCS in refinement is important, particularly at low resolution (Kleywegt, 1996 ▶), because it can drastically improve the effective data-to-parameters ratio.

*PROLSQ* (Hendrickson & Konnert, 1981 ▶) pioneered the use of structural superposition-based NCS restraints. This is where the two chains related by NCS are superposed and then restraints are used so as to pull each atom towards its NCS equivalent, thus reducing the superposition root-mean-square deviation. This approach has been adopted by most subsequent refinement programs, including *TNT* (Tronrud *et al.*, 1987 ▶), *X-PLOR* (Brünger, 1992*b* ▶), *CNS* (Brünger *et al.*, 1998 ▶) and *REFMAC* (Murshudov *et al.*, 2011 ▶). The *BUSTER* program (Bricogne & Irwin, 1996 ▶; Blanc *et al.*, 2004 ▶; Bricogne *et al.*, 2011 ▶) now uses superposition routines developed by Coutsias *et al.* (2004 ▶), which provide accurate gradient vectors, for its implementation of these restraints. Using superposition-based NCS restraints in practice proves to be laborious. Where electron density shows that residues have distinct conformations in different NCS copies, restraint lists have to be manually modified. Furthermore, for flexible multi-domain proteins it is often unclear how best to set up the different NCS relations required. This means that NCS is often not used when it could really help in the refinement of low-resolution structures and it is difficult to take advantage of it in automated refinement pipelines.

To provide easy-to-use automated NCS restraints, it was decided to adopt a different approach that uses interatomic distances rather than structural superposition, extending the ideas used in the *SHELX* program (Usón *et al.*, 1999 ▶). Local structural similarity restraints (LSSR) and the related -­autoncs and -target procedures have been developed and were incorporated in the *BUSTER* consortium release of July 2008 and in the academic *BUSTER* release of July 2009. The procedures have been described at a number of conference presentations (Smart *et al.*, 2008 ▶) and in the online *BUSTER* documentation. This paper presents the procedures in detail for the first time.

## 2. Using interatomic distances to provide similarity restraints

### 2.1. Local structural similarity restraints

NCS in haemoglobin structures will be used to illustrate the ideas behind using interatomic distances to provide restraints on molecular similarity. Haemoglobin was one of the first protein structures to be determined (Perutz *et al.*, 1998 ▶) and has been the subject of many structural studies, so that there are now around 180 PDB structures of haemoglobins from many sources and a wide variety of conditions. Haemoglobin exists as a tetramer of two α chains (normally given the chain identifiers A and C) and two β chains with a distinct sequence (chains B and D). Each of the four chains binds a haem prosthetic group that is involved in oxygen binding. The structure–function relationship of haemoglobin has been characterized in fine detail (Perutz *et al.*, 1998 ▶).

**Figure 1:**
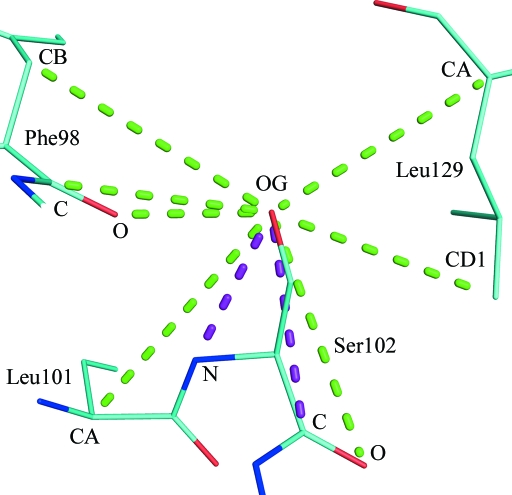
Haemoglobin 1y8k: selected close nonbonded (green dashes) and 1–4 contacts (purple dashes) of atom OG from SerA102. See also Table 1 ▶. All molecular-graphics figures were produced using *PyMOL* (DeLano, 2002 ▶).

PDB entry 1y8k (Sankaranarayanan *et al.*, 2005 ▶) is a well determined 2.3 Å resolution structure of horse methaemo­globin. Sankaranarayanan *et al.* (2005 ▶) state that in the final stages of refinement NCS restraints were not used. To illustrate the effect that NCS relations have on close nonbonded and 1–4 interactions, we will begin by looking at a selection of the contacts made by an arbitrarily chosen single atom: OG of SerA102 (Fig. 1 ▶). Table 1 ▶ compares the interatomic distances found in the A chain with those between the equivalent atoms in the NCS-related C chain. It can be noted that the distances in the two are similar but not identical. The absolute difference in interatomic distance can be used to gauge the differences, 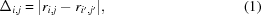
where *r*_*i*,*j*_ is the distance between atoms *i* and *j* in the A chain and *r*_*i*′,*j*′_ is the distance between the equivalent atoms in the NCS-related C chain. If the NCS between two chains is exact, so that the structure of the two chains is identical, then all Δ_*i*,*j*_ would be zero. Table 1 ▶ shows that the Δ_*i*,*j*_ for OG of Ser102 are nonzero, with four less than than 0.1 Å and the rest less than 0.4 Å.

**Figure 2:**
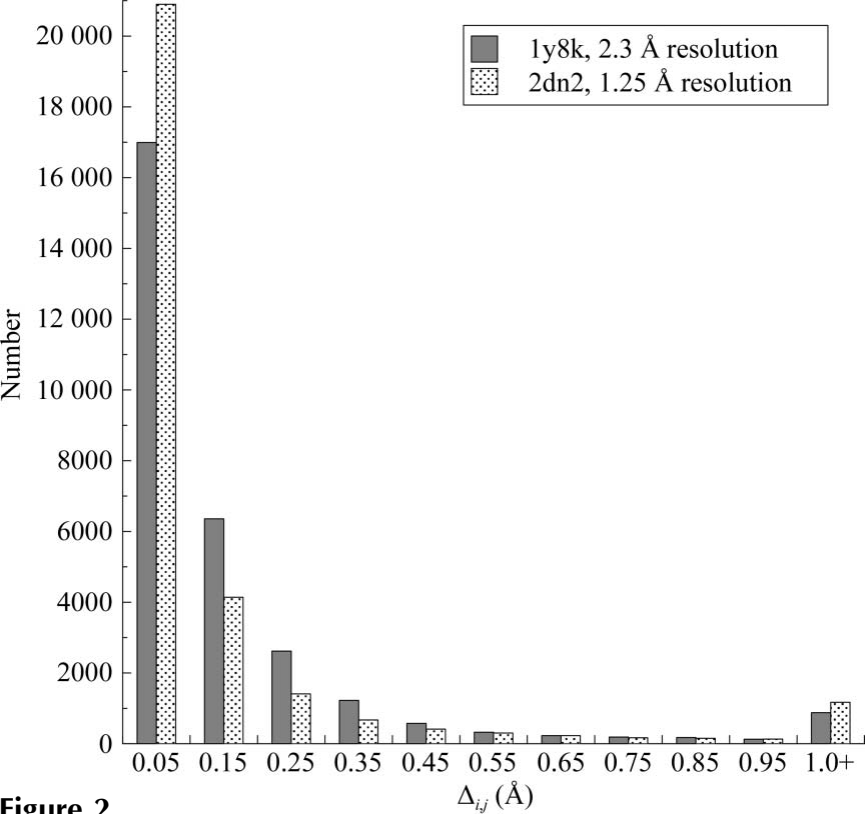
Distributions of the difference in close atomic distances for NCS-related atoms in two haemoglobin structures. Note that the comparison is between related chains within the same structure.

Instead of looking at individual distances and their differences, let us extend the analysis to all nonbonded atom pairs that are closer than 5.5 Å in the A chain or its NCS-related C chain. Contacts involving the haem groups are included in the analysis (but not water molecules). The analysis is further extended to include the equivalent atom pairs in the NCS-related B and D chains, including their haem groups. Fig. 2 ▶ is a histogram of the distribution found for the 29 600 Δ_*i*,*j*_ in the analysis. It can be noted that most Δ_*i*,*j*_ are in the first bin and so are less than 0.1 Å. This shows there is a high extent of NCS between related chains in haemoglobin, as would be expected. The histogram has a lengthy tail, with differences extending to 1 Å and beyond. This tail arises from moderate and large departures from NCS between related chains.

It is instructive to compare (Fig. 2 ▶) results for the ‘medium’-resolution structure 1y8k with the distribution of all Δ for the 1.25 Å resolution human oxyhaemoglobin structure 2dn2 (Park *et al.*, 2006 ▶). For the high-resolution structure the number of distances in the first bin (up to 0.1 Å) is higher. There are comparatively fewer Δ_*i*,*j*_ in the range 0.1–0.5 Å. However, above 0.5 Å the ‘tail’ has a similar population. It is unlikely that the difference in distributions reflects a genuine contrast in the degree of NCS similarity in the two haemoglobin structures. Instead, the smaller amount of X-ray data in the lower resolution structure means that the structures of each chain are less well determined, so that the NCS pairs diverge from one another to a greater extent.

**Figure 3:**
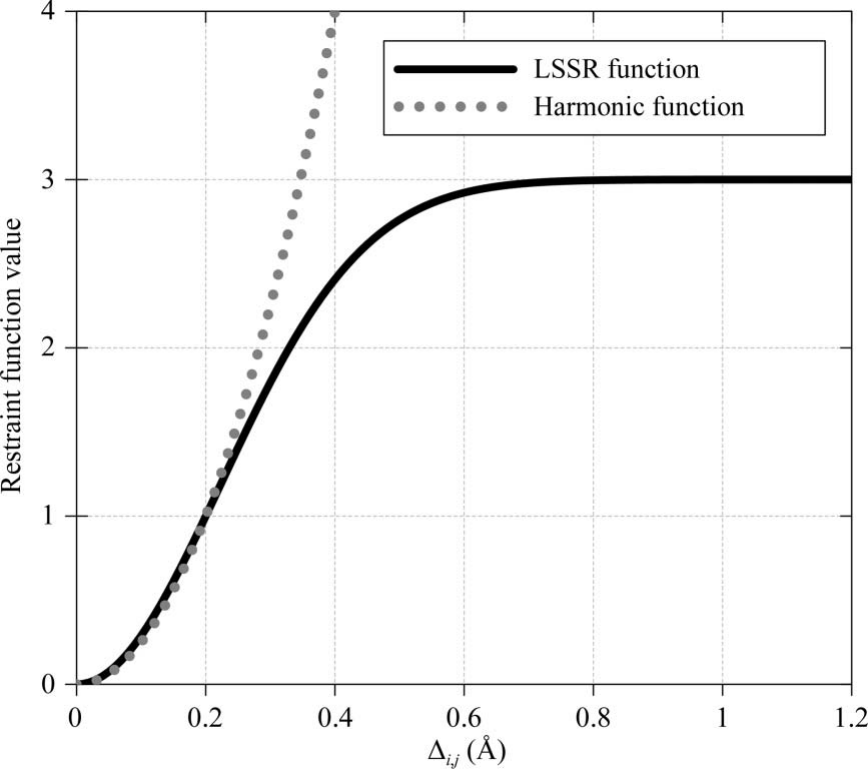
LSSR restraint penalty plateaus compared with a harmonic function.

A restraint that tightens the distribution of Δ below 0.5 Å, encouraging smaller values of Δ_*i*,*j*_, could be expected to be beneficial for the refinement of the medium-resolution structure. The tail observed in Δ_*i*,*j*_ above 0.5 Å represents genuine marked differences in the structures of NCS-related chains. Consequently, it would be a good thing for a restraint to apply a constant penalty in this region and so leave the differences unaltered. Because of this, it was decided to avoid using a harmonic functional form (Fig. 3 ▶). Instead, a function was chosen for LSSR to be close to harmonic below 0.2 Å but then to progressively level off so that it is flat for differences above 0.7 Å, 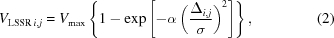
where Δ_*i*,*j*_ (the difference in NCS-related interatomic distances) is given in [Disp-formula fd1](1). The constant α is set so the function value equals 1.0 when Δ_*i*,*j*_ = σ, so 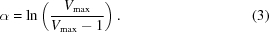
Restraint parameters of σ = 0.2 Å and *V*_max_ = 3 are used in practice, as these produce a restraint with the desired shape and have been found to yield good results. The resulting LSSR function is plotted in Fig. 3 ▶.

The total LSSR contribution to the *BUSTER* geometry function is found by applying the LSSR function (2) for all nonbonded atom pairs in related chains that are closer than 5.5 Å in either chain, 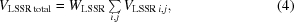
where *w*_LSSR_ is a weight that is adjusted in -autoncs (see below) or can be set by the user.

It should be noted that LSSR apply to the difference in related interatomic distances. This means that the restraints encourage related distances to be similar while not favouring any particular actual distance. The restraints encourage NCS-related chains to have similar local structure, but differences are allowed to occur at a fixed cost. The 5.5 Å distance cutoff was chosen to be as small as possible while ensuring that distances describing the geometry of interaction between hydrogen-bonding residues in α-helices and β-sheets are included.

LSSR involve producing a large number of individually rather weak restraints. For instance, for haemoglobin 29 600 restraints would be applied to the 5578 protein and haem atoms. As each restraint involves four atoms, this means that each atom is involved in an average of 21 LSSR restraints.

### 2.2. LSSR restraint setup and the –autoncs option

The current *BUSTER* implementation of LSSR and conventional superposition-based restraints requires that NCS-equivalent atoms have the same atom names and residue numbers but distinct chain identifiers. In the -autoncs option a comparison of residue name (such as ‘SER’) is made between residues with the same number (such as ‘102’) in distinct chains. LSSR are set up between two chains if more that 80% of residues with matching residue numbers have the same residue name.

For multiple-copy NCS, separate LSSR are set up coupling each pair of chains. For example, for threefold NCS chain *A* would have LSSR to chain *B* together with LSSR for chain *A* to chain *C* in addition to a separate set of LSSR coupling chain *B* and chain *C*. It was found in practice that this tended to overweight multiple-copy NCS. Accordingly, the -autoncs option now invokes a weight adjustment, 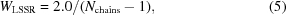
where *N*_chains_ is the number of chains related by NCS and *W*_LSSR_ is the LSSR weight that appears in (4). The weight adjustment produces good results in most cases, but if desired the user can specify *W*_LSSR_ explicitly.

**Figure 4:**
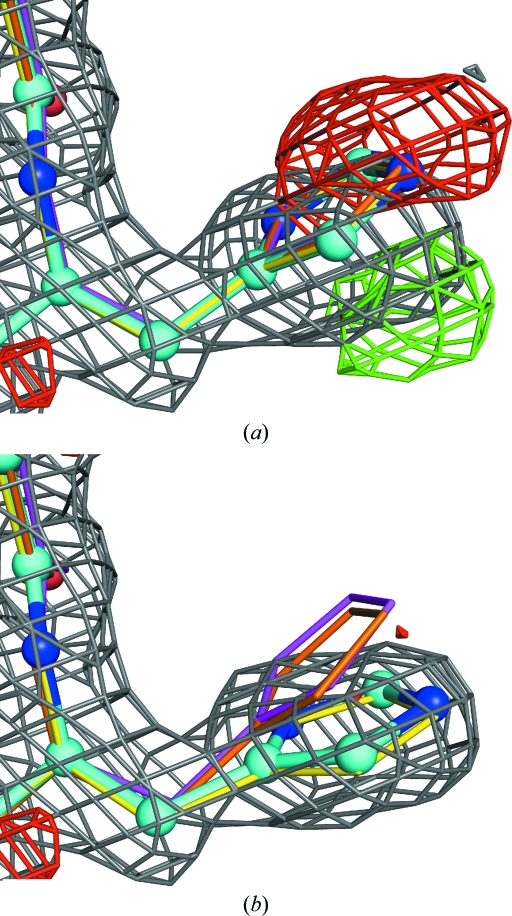
Re-refinement of PDB entry 3cmc demonstrates how automated LSSR pruning avoids model disruption where NCS copies have distinct but similar conformations. 3cmc is the 1.77 Å resolution structure of GAPDH determined by Moniot *et al.* (2008 ▶) with fourfold NCS. (*a*) shows how *BUSTER* refinement with the -autoncs_noprune option produces difference density (contoured at 3σ) close to His142 in the *P* chain (cyan with atom colouring). This is because LSSR forces a consensus conformation between the residue and its NCS equivalents, shown as superposed ‘ghosts’ in magenta, orange and yellow. (*b*) Using the -­autoncs option means that this residue is ‘pruned’ from the restraint lists and can adopt distinct conformations in the NCS copies nestling into the 2*F*_o_ − *F*_c_ density (shown in grey contoured at 1.2σ).

When using conventional superposition-based NCS restraints there is a need to edit or ‘prune out’ side chains or complete residues that are shown by the density to have distinct conformations in the chains related by NCS. Although the LSSR function reduces the need for restraint pruning by plateauing, it does not entirely eliminate it. Restraint-list pruning is still desirable for parts of the structure that are completely distinct in the NCS equivalents because some individual interatomic distances can be close by simple chance. In this case it is clearly not beneficial to couple these distances and so encourage them to be closer. Another rather more subtle situation also arises where the density indicates that parts of the structure are distinct in the NCS copies but the distinct copies are still rather similar. Fig. 4 ▶ demonstrates such a case.

The *BUSTER* -autoncs option provides automated LSSR list pruning. This is performed by initially setting up and calculating all LSSR restraints. The total LSSR function contribution is then found for each residue and compared with the maximum possible function contribution (if all LSSR involved were in the plateau region). If the ratio is above 0.5 then the residue has quite distinct environments between NCS copies and so all LSSR involving this residue are turned off. To identify residues that are distinct but similar in NCS copies, the average LSSR gradient is found for the residue. Large LSSR gradients are normally caused by the restraints ‘fighting’ the maximum-likelihood (ML) X-ray term (Fig. 4 ▶ *a*). This is a good indicator that the restraints are unhelpful and so the residues in question are removed from the LSSR list (Fig. 4 ▶ *b*). In practice, it is found that automated pruning can be unhelpful in the early rounds of refinement, in which case it can be turned off by using the -autoncs_noprune option instead of -autoncs. If desired, users can manually prune the LSSR list, but this is seldom necessary.

A common situation in NCS is that amino acids have similar conformations and environments in different NCS copies, but that equivalent atoms are labelled differently: consider a phenylalanine side chain that is similar in two NCS equivalents but for the fact that the labels of atoms CD1/CD2 and CE1/CE2 differ. Naive application of LSSR would wrongly regard the equivalent residues as having distinct conformations, leading to the disruption of similarity restraints in the region around them. A further degree of automation is therefore provided in the -sim_swap_equiv option to automatically swap equivalent atoms in the side chains of aspartic acid, glutamic acid, phenylalanine, tyrosine and arginine residues so as to increase the degree of similarity. Such swapping only changes the nomenclature for atoms that are equivalent. If desired, the swapping can be extended to include quasi-symmetric histidine, asparagine and glutamine residues by using the -sim_swap_equiv_plus option instead. In the extended case the procedure does involve physically swapping some non-equivalent atoms and can result in the disruption of hydrogen-bonding networks, so caution should be exercised if it is invoked.

In the current implementation of -autoncs water molecules are excluded from LSSR. This is because of the extant requirement that NCS-equivalent water molecules be supplied with identical residue numbers. This can be performed using the *CCP*4 (Winn *et al.*, 2011) program *SORTWATER* with subsequent manual LSSR setup in *BUSTER*. The *BUSTER* wiki (http://www.globalphasing.com/buster/wiki) includes an example of how to do this for PDB entry 4cha, a 1.68 Å resolution structure of chymotrypsin (Tsukada & Blow, 1985 ▶). It is found that using LSSR NCS restraints produces improvements in both *R*_free_ and the *R*_free_–*R*_work_ gap and that including water molecules in the restraints results in further small gains in these metrics.

It should be noted that the -autoncs option does not set up any restraint to couple isotropic temperature (‘*B*’) factors of atoms related by NCS. *TNT* and early versions of *BUSTER* used restraints to couple *B* factors when superposition-based NCS restraints were used. It is reasonably common to have chains that are related by NCS with a high degree of structural similarity but with a marked difference in temperature factors between the different chains. Although the *TNT* functional form for *B* coupling between NCS pairs allows for an offset in the average *B* of each chain without a penalty, it is found in practice that the restraints seldom result in any benefit in terms of *R*_free_. Accordingly, the -autoncs option does not activate them. Users can manually set up *B*-coupling NCS restraints and use them together with LSSR if desired.

### 2.3. LSSR to a target structure

The discussion so far has described how LSSR on inter­atomic distances can be used to restrain the molecular similarity found with NCS. The restraints can also be used for another commonly encountered case of molecular similarity, namely that to a separate already determined structure that remains fixed during the refinement of the structure being refined. We refer to the fixed structure as the ‘target’.

This situation can arise during drug-optimization ligand-soaking experiments where a high-resolution structure, possibly with a parent ligand compound, has already been determined. Soaking other compounds often involves using a disruptive solvent such as DMSO and can result in diffraction to a lower data resolution as well as in changes to unit-cell parameters. The original high-resolution structure is used as a molecular-replacement search model, but the conventional approach to the subsequent refinement would fail to further utilize the fact that the protein structure is in very many respects similar to the known high-resolution structure. For low data resolution, the situation can arise in which naive refinement from the MR solution can result in an *increase* in *R*_free_ (as shown below in §[Sec sec3.1]3.1). This indicates that better fitting the limited set of working data results in worsening the fit to the validation set, showing that information is being lost. We will show that using similarity restraints can prevent this loss.

The situation is analogous to NCS, except that instead of the similarity being between two chains within the structure under refinement it is between the complete protein structure and the fixed target. If the target and the structure being refined have the same space group and similar unit-cell parameters (as is common in soaking experiments) but different ligands, then the extent of similarity is likely to be greater than for a typical case of NCS. This is because the different chains in NCS have distinct packing environments, whereas in the soaking case the packing environments for most of the protein will be similar in the two structures.

This analogy prompted us to adapt the restraints developed for NCS to the treatment of similarity to fixed target structures. The initial *BUSTER* implementation of this approach (which predated LSSR) was to adapt conventional superposition-based NCS restraints for target similarity (Malet *et al.*, 2007 ▶). In the refinement of a 3.0 Å resolution structure of the RNA polymerase domain of West Nile virus nonstructural protein 5, using the method with a higher (2.35 Å) resolution target structure allowed a ‘stalled’ process of refinement and model building to be resumed, contributing to a drop in *R*_free_ of 2.8% (Malet *et al.*, 2007 ▶). However, the approach involved a manually intensive process of producing separate domain definitions and a long list of similarity exceptions for residues and side chains that have distinct conformations in the two structures, as described in detail in Malet *et al.* (2007 ▶).

To provide a more user-friendly approach, LSSR have been adapted so as to generate restraints to a target structure. *BUSTER* includes a routine to read one or more target structures in PDB format. Normally, target LSSR assumes that an atom in the structure under refinement is related to an atom in the target structure with the same atom name, residue number and chain identifier. Equation (1) is used to find the difference in interatomic distances for close contacts between equivalent atoms in the refined structure (*r*_*i*,*j*_) and in the fixed target structure (*r*_*i*′,*j*′_). The *BUSTER* command-line option -­target related.pdb provides an easy-to-use method for specifying the target-structure PDB file and activating LSSR to it.

### 2.4. Comparison between LSSR and other techniques

LSSR had its origins from strong user feedback that the use of conventional superposition-based NCS restraints in *BUSTER* was far too complicated for routine use. Discussions on the CCP4 bulletin board praised the *SHELX* method of using differences in 1–4 distances as the basis for NCS restraints (Usón *et al.*, 1999 ▶), in particular in that it overcomes the need for defining separate domains. However, considering only 1–4 distances seemed limited as it could not favour similarity in ligand–protein contacts or between disconnected β-sheet strands. LSSR use close interatomic distances in addition to 1–4 distances to overcome these limitations. It can be noted that *X-PLOR* provides ‘distance symmetry restraints’ to impose similarity between two or more chains in NMR structure determination (Brünger, 1992*b* ▶) through a harmonic penalty term. *X-PLOR* distance symmetry restraints are normally applied to the distances between all pairs of C^α^ atoms, with the result of strictly enforcing similarity (Brünger, 1992*b* ▶). In contrast, LSSR are short-range and use a non­harmonic functional form to allow true deviations from similarity. This idea was inspired by the distance restraints used in the *MODELLER* program for homology information (Sali & Blundell, 1993 ▶) and by NOE restraints used in NMR structure refinement in *X-PLOR* (Brünger, 1992*b* ▶) and *CNS* (Brünger *et al.*, 1998 ▶). In both cases, restraints favour a particular distance but allow this to be violated at a fixed cost. In LSSR the use of a functional form that plateaus at large values also means that there is a limit on the penalty applied to large violations.

The idea of using restraints to a target structure has its origin in harmonic restraints to initial positions commonly used in the initial stages of molecular-dynamics simulations (McCammon & Harvey, 1988 ▶). *X-PLOR* (Brünger, 1992*b* ▶) allows the use of ‘point restraints’ to harmonically restrain the coordinates under refinement to specific points in space from a reference coordinate set. The Deformable Elastic Network (DEN) method was developed by Brunger and coworkers (Schröder *et al.*, 2007 ▶, 2010 ▶) for the simulated-annealing refinement of low-resolution structures. DEN uses restraints from higher resolution structures or electron microscopy. Harmonic restraints on close interatomic distances are used. Where the data require deviations these are enabled by a gradual resetting of the restraint ideal values during the simulated-annealing process. In developing LSSR, we chose to use a restraint form that plateaus and some list pruning rather than a gradual reset process, as it better suits an optimization-based refinement procedure. To date, the focus of the DEN method has been the solution of new low-resolution structures (Schröder *et al.*, 2010 ▶) rather than the refinement of protein–ligand complexes.

LSSR for NCS and target applications share many features with the ‘local NCS’ and ‘External structure restraints’ recently introduced into *REFMAC* (Murshudov *et al.*, 2011 ▶). These were developed independently from LSSR at much the same time. Differences in close interatomic distances are used, together with a different plateauing-function form. The *REFMAC* implementation uses sequence alignment to find equivalent parts of chains, avoiding the need for the prior assignment of residue numbers. *REFMAC* also provides for the easier inclusion of water molecules in NCS (Murshudov *et al.*, 2011 ▶). *BUSTER* appears to have advantages in the automation of restraint pruning and in side-chain flipping.

## 3. Example applications

### 3.1. Solving a low-resolution complex of RNAse T1 using –­targetrestraints

Lenz *et al.* (1991 ▶) published the structure of ribonuclease T1 (RNAse T1) with the nucleotide guanosine-3′,5′-bisphosphate (pGp) bound. The structure was determined from an in­complete (90%) 3.2 Å resolution room-temperature data set collected on a four-circle diffractometer with a sealed-tube source. The structure was determined by MR and refined using the least-squares refinement program *PROFFT*. As well as the ligand, 89 water molecules were included in the structure. The structure and structure factors were deposited and are available as PDB entry 5rnt. The structure was determined before the *R*_free_ procedure was proposed (Brünger, 1992*a* ▶) and before ML refinement procedures were available. Given the low data resolution, this led to overfitting and phase-bias problems.

The same group later determined the structure of RNAse T1 with pGp bound at a much higher (1.8 Å) resolution (Lenz *et al.*, 1993 ▶). Compared with the low-resolution 5rnt structure the crystals were in the same *I*23 space group, with only a small difference in unit-cell dimension. The pGp ligand-binding position differed from the previous low-resolution result, particularly in the positioning of the guanine ring. In addition, a phosphate anion was found to be bound in the catalytic site that had not been observed in the low-resolution structure. The high-resolution structure is not available in the PDB.

PDB entry 5rnt provides an interesting test case showing that contemporary methods can yield useful information for this low-resolution data set, particularly when target LSSR are used. The descriptions given by Lenz *et al.* (1993 ▶) provide a guide to the expected ligand and phosphate-binding positions in RNAse T1–pGp. Accordingly, it was decided to re-solve RNAse T1–pGp.

The best MR search model now available is PDB entry 1det, a 1.95 Å resolution RNAse T1 structure (Ishikawa *et al.*, 1996 ▶) with the same *I*23 space group as 5rnt and a similar unit-cell dimension. 1det has a guanosine 2′-phosphate (2′GMP) nucleotide bound and the RNAse T1 is covalently modified by carboxylmethylation of the active-site residue Glu58. In using LSSR target restraints it is sensible to ensure that the high-resolution target structure has as good a structure as possible. Consequently, 1det was first re-refined and rebuilt (see Supplementary Material^1^). The rebuilding improved the fit to the data and the geometry of the protein, as assessed by *MolProbity* (Chen *et al.*, 2010 ▶; see Supplementary Material). In the original 1det structure the 2′GMP ligand was found to have a chiral inversion at the 2′ carbon and this is corrected in the rebuilt structure (see Supplementary Material). The rebuilt 1det model has been deposited in the PDB and has been assigned PDB code 3syu.

To re-solve RNAse T1–pGp, the structure factors for 5rnt were obtained from the PDB (Berman *et al.*, 2000 ▶). The *CCP*4 (Winn *et al.*, 2011 ▶) program *CAD* was employed to transfer the previously assigned free set of reflections from the rebuilt 1det structure and apply it to the 5rnt structure factors. It is important to do this when using LSSR targeting with the same cell and space group to avoid any possibility of free-set contamination. The *CCP*4 (Winn *et al.*, 2011 ▶) program *MOLREP* (Vagin & Teplyakov, 2010 ▶) was used to find an MR solution with structure factors from 5rnt. The MR search model was based on the rebuilt 1det structure stripped of ligands, carboxylmethylation, H atoms and water molecules. Residue 25 was altered from a Gln to a Lys, as this residue differs in the two proteins. *MOLREP* found a clear solution with a high contrast and an *R* value of 0.33. The *MOLREP* solution agreed with 5rnt as to placement of the protein within the unit cell.

**Figure 5:**
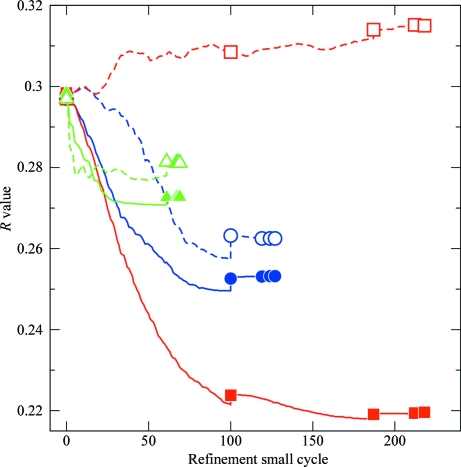
Initial *BUSTER* refinements of the RNAse T1–pGp MR structure. Solid lines with filled symbols indicate *R*_work_. Dashed lines with open symbols indicate *R*_free_. The standard refinement is shown in red with squares. Refinement with target LSSR is shown in blue with circles and the control rigid-body refinement is shown in green with triangles.

Fig. 5 ▶ compares different protocols for the initial ML refinement of the MR solution with *BUSTER* (Bricogne *et al.*, 2011 ▶). In all cases the standard *BUSTER* objective function consisting of an ML X-ray function plus stereochemical restraints on bonds, angles, torsions, planes and ideal contacts was used. In addition, individual atomic temperature factors are allowed to vary but with stiff harmonic restraints coupling the *B* factors of bonded atoms.

The initial run is a standard *BUSTER* refinement where all atoms are allowed to move with no additional restraints or constraints to exploit similarity. Fig. 5 ▶ shows that in this case there is a rapid decrease in *R*_work_ but that *R*_free_ increases compared with the starting value. The standard refinement also significantly degrades the *MolProbity* geometry measures (Table 2 ▶). *MolProbity* provides a overall score that approximates to a nominal resolution of the structure. In this case the overall score for the initial MR model is 0.86 Å, reflecting the ‘perfect’ geometry of the rebuilt 1det structure. Conventional *BUSTER* refinement degrades the *MolProbity* overall score to 2.24 Å, introducing four bad side-chain rotamers and moving four residues from Ramachandran favoured regions. The increase in *R*_free_ and the degradation of the geometry metrics reflect that the refinement has too many soft degrees of freedom for the small number of X-ray reflections in the low-resolution data set. The refinement overfits the *R*_work_ data and the validation data in *R*_free_ indicate that information is being lost from the initial MR solution.

In contrast, *BUSTER* refinement with target LSSR to the rebuilt 1det structure results in a marked decrease in *R*_free_. In addition, the gap between *R*_free_ and *R*_work_ is kept to around 1%, in contrast to the standard run with a wide 9.6% gap (Table 2 ▶). *MolProbity* protein geometry metrics remain almost ‘perfect’ in the target run (Table 2 ▶) instead of degrading. The target LSSR allow the refinement to exploit the information that the structure of the protein will in many respects be similar to that determined for the higher resolution protein–ligand complex model. The restraints allow the protein to move when the X-ray data or short crystal contacts demand it but provide a penalty for changing parts of the structure to fit noise in the X-ray term.

A control for the use of target LSSR is to use rigid-body refinement. Here, the structure of the protein is kept fixed to that of the high-resolution structure with only six positional degrees of freedom allowed: displacement and rotation of the rigid protein. Temperature factors are allowed to vary but are coupled with stiff harmonic restraints. Fig. 5 ▶ shows that this approach is an improvement over the standard run, with no decrease in *R*_free_. However, *R*_free_ remains above that found with target LSSR. Rigid-body refinement enforces exact similarity by allowing no freedom for the protein to change to fit to the density. It formally reduces the number of parameters to be optimized in the fit drastically. This results in a faster initial drop in *R*_free_ compared with that found with target LSSR (Fig. 5 ▶). For this reason, *BUSTER* has an option to apply an initial round of rigid-body refinement that is recommended for use when refining from an MR solution. The problem with a rigid-body approach is that it precludes any structural change within the rigid body, leaving poor geometry at crystal contacts and preventing movements even where maps clearly indicate that change is needed. The usual solution to this is to exclude parts of the protein from the rigid body, allowing them full positional freedom. This approach has been used for the refinement of low-resolution structures (ter Haar *et al.*, 2007 ▶) but is laborious in practice. Target LSSR provide a much more convenient method, exploiting similarity while allowing change without altering rigid-body definitions.

**Figure 6:**
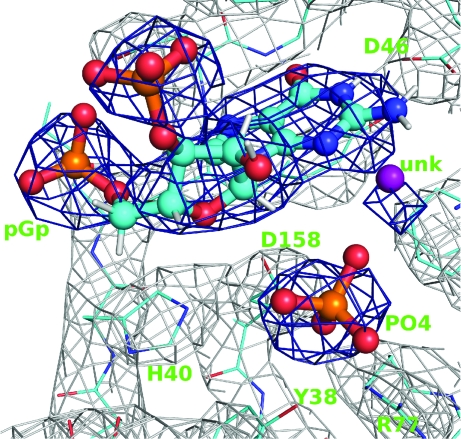
The active site of the final RNAse T1–pGp model. The pGp ligands, phosphate anion and an unknown cation or water molecule are emphasized with sticks and spheres. 2*F*_o_ − *F*_c_ density is contoured at 1.2σ and shown in grey for the protein and dark blue around the ligands.

Examination of the difference density following initial *BUSTER* refinements showed that the rigid-body control had peaks near the protein where the data indicated that small protein movements were necessary. Other than this, the difference maps were similar for the three initial refinements, with clear difference density for the pGp ligand found close to the active site. Because of the better refinement statistics (Table 2 ▶) the model from initial refinement using target LSSR was used for subsequent building. A restraint dictionary for pGp was produced using the *grade* program (Smart *et al.*, 2011 ▶) based on data obtained from the CSD database using the *Mogul* program (Bruno *et al.*, 2004 ▶). Positioning the pGp ligand with *rhofit* (Womack *et al.*, 2010 ▶) and subsequent refinement (with target LSSR) strengthened clear density for a separate tetrahedral anion in the catalytic site. Following Lenz *et al.* (1993 ▶) this was modelled as a phosphate (Fig. 6 ▶). Clear density for a water molecule or small anion was found lying between the phosphate and the guanine ring of pGp (Fig. 6 ▶). Difference density peaks above 3σ were then observed at the positions occupied by eight water molecules in the rebuilt 1det structure. Water molecules were added to the rebuilt model at these positions with consistent residue numbering so that their positions were restrained by target LSSR in the subsequent refinement round. Adding these water molecules lowered the *R*_free_ by 0.2%, supporting their inclusion in the model, despite the fact that little 2*F*_o_ − *F*_c_ density was found for them.

The pGp ligand conformation, its binding contacts and the positioning of the phosphate anion in the catalytic site (Fig. 6 ▶) are consistent with those described by Lenz *et al.* (1993 ▶) for the same complex solved at 1.8 Å resolution (see Supplementary Material^1^). It can be concluded that *BUSTER* ML refinement with target LSSR allows the most important features of the pGp T1 RNAse complex to be found from low-resolution data.

Final refinement and geometry statistics for the rebuilt 5rnt model are given in Table 3 ▶. Comparison is made to the results of a control refinement in which all solvent molecules were stripped from the original 5rnt model and it was subjected to a long standard *BUSTER* refinement with the same *grade* dictionary for pGp. It can be seen that careful rebuilding of 1det and then 5rnt results in a structure with an *R*_free_ 7% lower than the control and very much better *MolProbity* statistics. The rebuilt 5rnt model has been deposited in the PDB and has been assigned PDB code 3urp.

### 3.2. Re-refinement of PDB entry 1osg: the –autoncs option contributes to finding an extra copy of the ligand

The usefulness of LSSR on NCS through the -autoncs option is demonstrated in the re-refinement of PDB entry 1osg (Gordon *et al.*, 2003 ▶), a 3.0 Å resolution structure of the tumour necrosis factor protein BAFF. In 1osg the protein is complexed with bhpBR3, a 12-residue β-hairpin peptide containing a six-residue turn from the BR3 receptor that forms the binding region for BAFF in signalling. The bhpBR3 peptide is cyclized by the formation of a disulfide bond between cysteine residues at its N- and C-termini. The β-­hairpin structure of isolated bhpBR3, determined by solution NMR (Kayagaki *et al.*, 2002 ▶), is maintained in the BAFF complex 1osg (Gordon *et al.*, 2003 ▶). The 1osg structure is composed of two BAFF trimers related by a twofold NCS axis. Each of the protein subunits binds a bhpBR3 peptide. Consequently, both the protein and its ligand have sixfold NCS. The 1osg structure is well built and was originally refined with *REFMAC* using conventional superposition-based restraints on NCS, except for BAFF residues 215–226, for which distinct conformations between NCS equivalents were reported (Gordon *et al.*, 2003 ▶).

The 1osg structure and structure model were downloaded from the PDB (Berman *et al.*, 2000 ▶) and stripped of water molecules and magnesium ions. The structure was then subjected to an initial *BUSTER* refinement in which TLS parameters together with individual restrained *B* factors were refined, but the atomic coordinates were kept fixed. 12 TLS groups were used, one for each protein and peptide chain. Table 4 ▶ shows that the adjustment of temperature factors results in a substantial (1.6%) drop in *R*_free_. From this position, a series of further *BUSTER* refinements assessed the effect of positional refinement with different approaches to NCS restraints (Table 4 ▶). Standard *BUSTER* procedures and weights were used for all runs. The -sim_swap_equiv_plus option (described in §[Sec sec2.2]2.2) was used in refinements with NCS restraints in order to to automatically swap equivalent atoms in side chains to improve the degree of NCS similarity between the chains (around 49 out of 922 residues were adjusted by the procedure). The runs with superposition-based (r.m.s.d.) NCS restraints used a manually written control file with an NCS restraint σ of 0.1 Å.

A control *BUSTER* refinement without any NCS restraints resulted in a small drop in *R*_free_ and an improvement in the *MolProbity* geometry score but with a considerable opening of the *R*_free_–*R*_work_ gap (Table 4 ▶). All refinements using NCS restraints produce drops in *R*_free_, narrow the *R*_free_–*R*_work_ gap and give improvements in the *MolProbity* geometry score compared with the PDB model. However, the naive application of superposition-based NCS to the whole structure results in considerable disruption to the PDB model, pulling the loop 215–226 from the carefully modelled conformations found in 1osg (Gordon *et al.*, 2003 ▶) and resulting in large difference density features. The disruption is reduced, but not eliminated, when r.m.s.d. NCS restraints are used with the loop removed. Minimal disruption and the best *R*_free_ are found with the -­autoncs output (Table 4 ▶). The -autoncs procedure leaves alone side chains that have been modelled into density. Consequently, it provides the benefit of NCS restraints without having to work out NCS exception lists manually.

**Figure 7:**
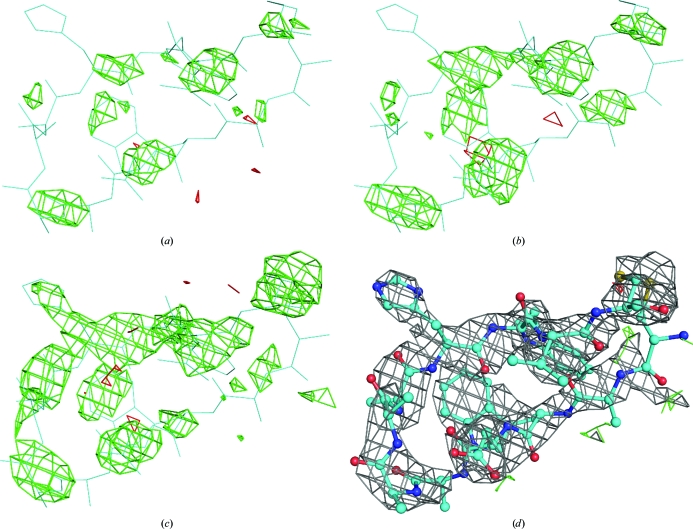
*BUSTER* refinement with -autoncs and TLS strengthens density for an additional copy of the cyclized peptide bhpBR3 ligand. (*a*) shows *EDS* (Kleywegt *et al.*, 2004 ▶) difference density in the region. *BUSTER* difference density for the 1osg PDB model with no further refinement is shown in (*b*). Refinement using -autoncs and TLS strengthens the difference density (*c*), allowing the identification of an additional binding site for bhpBR3. (*d*) shows the *Z*-chain bhpBR3 peptide from the final refined model together with 2*F*_o_ − *F*_c_ density contoured at 1.0σ. Difference density is contoured at 3.0σ in all cases. It should be noted that no peptide was included in the map calculation or refinement for (*a*), (*b*) and (*c*): the cyan wire frame for the peptide is a ‘ghost’ of the model from (*d*).

Taken together, the use of *BUSTER* TLS refinement together with -autoncs produces a 3.9% reduction in *R*_free_ compared with the Gordon *et al.* (2003 ▶) model and narrows the *R*_free_–*R*_work_ gap while improving the *MolProbity* geometry scores (Table 4 ▶). These improvements are a good thing in themselves, but the more important consequence is that the improved modelling of the structure reveals new features in the difference density that allow additional molecular detail to be built. In particular, difference density appears that indicates the presence of an additional (seventh) copy of the cyclic bhpBR3 peptide (not modelled in 1osg) in the structure (Fig. 7 ▶ *c*).

**Figure 8:**
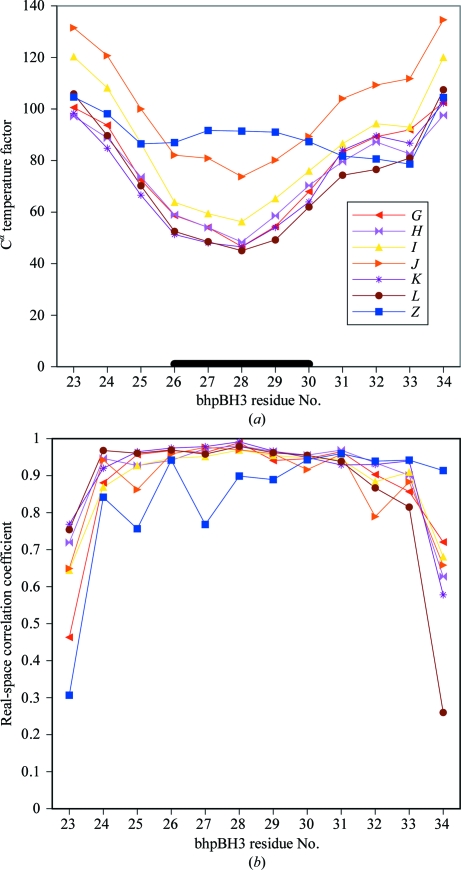
The temperature factors and main-chain correlation coefficients for the bhpBR3 peptides in the final remodelled 1osg structure. (*a*) shows the isotropic equivalent *B* factor for the α carbon. The six-residue loop from BR3 that is recognized by BAFF is marked by a thick black line. (*b*) shows the correlation coefficient to 2*F*_o_ − *F*_c_ for main-chain atoms. The high *B* factors and poor correlation for the N- and C-terminal cysteine residues are likely to arise from radiation damage to their disulfide.

To confirm that the density is for an additional bhpBR3, the peptide was modelled into the site using *Coot*. The *K*-chain copy of bhpBR3 from the -autoncs refined structure was duplicated, assigned the *Z*-chain identifier, stripped of its side chains (apart from the cystine) and fitted as a rigid body to the difference density. Further *BUSTER* refinement produced difference density in the expected positions for five of the missing side chains. These side chains were modelled using *Coot* and further refined with *BUSTER*. In the final model, the additional *Z*-chain copy bhpBR3 (Fig. 7 ▶ *d*) has real-space correlation coefficients that are close to those for the original six copies of the peptide in the structure (Fig. 8 ▶ *a*). The C^α^ temperature factors for the additional peptide are comparable to the original, but do not show the dip for the loop that binds to BAFF (Fig. 8 ▶ *b*).

The *Z*-chain copy of bhpBR3 is located at a lattice contact lying between three different asymmetric units. The peptide forms two main chain–main chain parallel β-sheet-type hydrogen bonds to the *K*-chain copy of bhpBR3. The two hydrogen bonds link peptides that are involved on the other sides in intramolecular β-sheet-type hydrogen bonds. The two copies of the peptide therefore join to form a small β-sheet. Residues His31 and Trp32 of the *Z*-chain peptide form hydrogen bonds to BAFF across lattice contacts. The fact that the extra copy of the bhpBR3 is located at a lattice contact means that it has no importance in the biological activities of BAFF. However, it does show that ‘dissected’ peptides can form such accidental contacts, implying that care must be taken to avoid the overinterpretation of structural features.

To see why the extra copy of the peptide was not observed by Gordon *et al.* (2003 ▶), it is instructive to examine the difference density in this region (Fig. 7 ▶). The *EDS* server (Kleywegt *et al.*, 2004 ▶) uses *REFMAC* to calculate maps for PDB entries and so provides a plausible representation of the final maps as examined by Gordon *et al.* (2003 ▶). The *EDS* map shows patches of disconnected density in the region (Fig. 7 ▶ *a*). The *BUSTER* map for the unrefined 1osg model (Fig. 7 ▶ *b*) strengthens the density but it still would not be interpretable. The use of *BUSTER* TLS refinement together with -autoncs connects the density in such a way that the β-hairpin becomes clearly visible (Fig. 8 ▶ *d*). Density for the extra peptide is also improved in maps from the *PDB_REDO* server (Joosten *et al.*, 2009 ▶), which uses *REFMAC* refinement including TLS and NCS restraints, but is not as clear as the *BUSTER* results.

**Figure 9:**
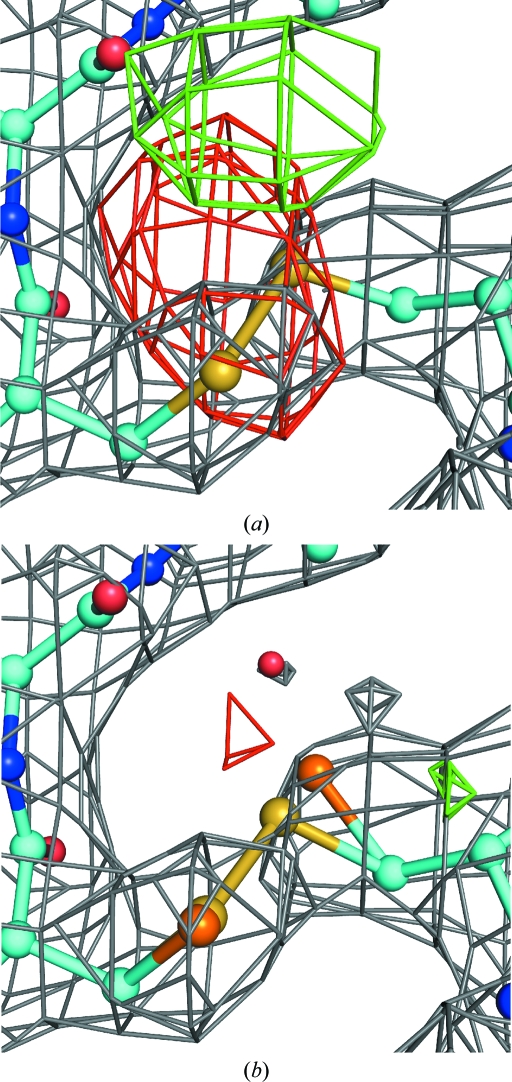
Difference density at the BAFF disulfide between residues 232 and 245. The pictures are for the *B* chain, but the other NCS copies are similar. (*a*) *BUSTER* refinement of 1osg with TLS and NCS produces an 8σ negative difference density peak. (*b*) A much better fit to density is found by modelling partial disulfide-bond formation using two alternates for the Cys232 and Cys245 SG atoms. Shown in yellow is alternate ‘*A*’ with a disulfide bond between the atoms. The alternate ‘*B*’ marked in orange has the S atoms in a reduced form. A bound water molecule that forms three good hydrogen-bond contacts can be placed nearby. The 2*F*_o_ − *F*_c_ density is contoured at 1.2σ and the *F*_o_ − *F*_c_ difference density at 3.5σ.

The largest difference-map features after *BUSTER* refinement of 1osg are negative peaks found at the disulfide between residues 232 and 245 of the BAFF protein (Fig. 9 ▶ *a*). Peaks are found at all six NCS-related sites with a magnitude of −7σ to −9σ. The peaks indicate that the density is not compatible with a fully formed disulfide bond. One possibility is that disulfide-bond formation in the BAFF protein was incomplete at the protein production and purification stage (Hymowitz, 2011 ▶). An alternative is that the effect is a consequence of radiation damage to the disulfide bond during data collection (Burmeister, 2000 ▶; Weik *et al.*, 2000 ▶). Gordon *et al.* (2003 ▶) state that the X-ray data collection resulted in a 3.5-­fold data redundancy. It would be very interesting to know the results of reprocessing of the diffraction images and of using only data collected in the initial stages of data collection: this would make it possible to distinguish between radiation damage and initial partial disulfide-bond formation.

To model the effect of either radiation damage or incomplete disulfide formation, the final remodelled 1osg structure has two alternates for the Cys SG atoms (Fig. 9 ▶ *b*). In the first alternate the atoms form a disulfide. In the second alternate the atoms are unbound in a reduced form. The occupancies of the alternates is allowed to vary during refinement. To allow the possibility that the S atom disappears owing to radiation damage no restriction is placed on the total occupancy for the SG atoms. To avoid adding too many parameters in refinement, the occupancies of all NCS-equivalent SG atoms are set to be identical. This model markedly reduces the amount of difference density in the region (Fig. 9 ▶ *b*) in addition to improving *R*_free_. The refinement results in an occupancy of 0.20 for the disulfide alternate, 0.57 for the reduced form of Cys232 and 0.51 for the reduced form of Cys245. This implies that approximately 25% of the S atoms have ‘disappeared’ owing to radiation damage, although initial partial disulfide formation cannot be ruled out.

Weik *et al.* (2000 ▶) have shown that radiation damage can completely break disulfide bonds and remove density for the S atoms. Solvent-exposed disulfide bonds are found to be more vulnerable to radiation damage and this damage is normally accompanied by an increasing loss of higher resolution data with exposure (Weik *et al.*, 2000 ▶). Radiation-damage changes can be exploited as a source of phase information (Schiltz & Bricogne, 2007 ▶). Although the disulfide bonds in BAFF lie at the centre of the protein trimer, there is indication of a bound water molecule close to each one and a large cavity next to this. Although the disruption to the disulfide in BAFF is distant from the bhpBR3 ligand, it is important to note that the ligand is held in its β-hairpin conformation by a disulfide bond and that this disulfide is completely solvent-exposed in the 1osg structure. The N- and C-terminal cysteine residues in the seven copies of bhpBR3 are characterized by high *B* factors and poor real-space correlation coefficients (Fig. 8 ▶). It is possible that this is simply because this part of the peptide lies furthest from the protein and is more mobile. However, alternatively the effect could arise from radiation damage breaking the disulfide bond in the ligand.

The rebuilt 1osg model with the extra copy of the peptide, partial disulfide model and other small improvements in the structure further benefits *R*_free_, *R*_work_ and *MolProbity* scores (Table 4 ▶). The final model has been deposited in the PDB and has been assigned PDB code 3v56.

## 4. Concluding remarks

This study demonstrates that for low-resolution structures the judicious use of prior information either from previous high-resolution structures or from NCS restraints can give useful benefits and can make a difference to the investigator’s ability to model the critical features of a structure. The fact remains, however, that a low-resolution structure is a low-resolution structure. It is important to remember that a good (less than 20%) *R*_free_ for a 3.0 Å resolution structure means rather less than the same metric for a 2.0 Å resolution structure. Much fewer data are involved and the detailed features of a structure will therefore tend to be more poorly defined.

The authors of the protein structures re-examined here (Lenz *et al.*, 1991 ▶; Ishikawa *et al.*, 1996 ▶; Gordon *et al.*, 2003 ▶) deposited structure-factor data as well as the protein structures (this was optional at the time). Without this, it would have been impossible to make the improvements described here. The PDB should also be congratulated for facilitating the deposition of re-examinations of existing PDB entries by the ‘REMARK 0’ re-refinement notice (used for the three depositions resulting from this work). The process enables corrections to be made to existing structures when new techniques reveal additional details or when problems are found. In conjunction with projects such as *PDB_REDO* (Joosten *et al.*, 2009 ▶), the deposition of re-refined protein structures provides a mechanism for the database of protein structures to be made more useful, in particular for nonspecialist users (Velankar & Kleywegt, 2011 ▶).

## Supplementary Material

PDB reference: RNAse T1, 3syu

PDB reference: RNAse T1–pGp, 3urp

PDB reference: BAFF, 3v56

Supplementary material file. DOI: 10.1107/S0907444911056058/ba5178sup1.pdf

## Figures and Tables

**Table 1:** Haemoglobin 1y8k: comparison of distances for NCS equivalents of the contacts in Fig. 1 ▶

Atom *j*	*r*_A102 OG,* j*_ (Å)	Atom *j*′	*r*_C102 OG, *j*′_ (Å)	Δ_A102 OG,* j*_ (Å)
A102 N	2.912	C102 N	3.000	0.09
A102 C	3.765	C102 C	3.724	0.04
A102 O	4.550	C102 O	4.427	0.12
A101 CA	5.102	C101 CA	5.205	0.10
A98 O	2.671	C98 O	3.051	0.38
A98 C	3.783	C98 C	4.180	0.40
A98 CB	5.056	C98 CB	5.367	0.31
A129 CA	4.623	C129 CA	4.273	0.35
A129 CD1	4.314	C129 CD1	3.918	0.40

**Table 2:** Initial *BUSTER* refinements of the RNAse T1–pGp MR structure

	Standard	Target LSSR	Rigid body
*R*_work_	0.220	0.253	0.273
*R*_free_	0.315	0.263	0.281
100(*R*_free_ − *R*_work_) (%)	9.6	0.9	0.8
*MolProbity* overall score (Å)	2.24	0.87	0.87
*MolProbity* bad rotamers	4/84	0/84	0/84
*MolProbity* Ramachandran outliers	1/101	0/101	0/101
*MolProbity *Ramachandran favoured region	96/101	99/101	99/101

**Table 3:** Final refinement and geometry statistics for the rebuilt 5rnt model

	5rnt PDB	Control 5rnt re-refined	Rebuilt 5rnt
*BUSTERR*_work_	0.2318	0.1750	0.2018
*BUSTERR*_free_	N/A	0.3066	0.2372
100(*R*_free_ − *R*_work_) (%)	N/A	13.2	3.5
*MolProbity* overall score (Å)	3.53	2.87	0.92
*MolProbity* clashscore	22.80	11.40	1.35
*MolProbity* bad rotamers	23/85	10/85	1/85
*MolProbity* Ramachandran outliers	2/102	2/102	0/101
*MolProbity* Ramachandran favoured region	90/102	94/102	99/101
R.m.s. bond-length deviation (Å)	0.018	0.010	0.007
R.m.s. bond-angle deviation (°)	3.90	1.27	1.00
*MolProbity* residues with bad angles (%)	20.2	0.0	0.0
pGp correlation coefficient	0.8715	0.9258	0.9171

**Table 4:** *BUSTER* re-refinements of 1osg

Conditions	*R*_work_	*R*_free_	100(*R*_free_ − *R*_work_) (%)	*MolProbity* overall score (Å)
1osg PDB entry	0.192	0.249	5.7	2.81
Initial TLS/*B* factor only (‘Init’)	0.178	0.233	5.5	2.81
From ‘Init’, no NCS restraints	0.159	0.232	7.4	2.51
From ‘Init’, r.m.s.d. NCS restraints on all atoms	0.180	0.215	3.5	2.24
From ‘Init’, r.m.s.d. NCS restraints except loop 215–226	0.176	0.212	3.6	2.31
From ‘Init’, -autoncs_noprune	0.172	0.211	3.8	2.23
From -autoncs_noprune, -autoncs	0.170	0.210	4.0	2.24
Final rebuilt model	0.162	0.200	3.8	1.83

## Footnotes

1 Supplementary material has been deposited in the IUCr electronic archive (Reference: BA5178). Services for accessing this material are described at the back of the journal.
