# Supplementary material for: Exploiting structure similarity in refinement: automated NCS and target-structure restraints in BUSTER
Source: Acta Crystallogr D Biol Crystallogr. 2012 Mar 16;68(Pt 4):368–80. doi: 10.1107/S0907444911056058 (PMC3322596; doi:10.1107/S0907444911056058)
Supplement: Supplementary file 1 [file d-68-00368-sup1.pdf]

Acta Cryst. (2012) D68, XXX–XXX

Supplementary materials:

Exploiting structure similarity in refinement: automated NCS and target structure restraints in *BUSTER*

Oliver S. Smart, Thomas O. Womack, Claus Flensburg, Peter Keller, Wlodek Paciorek, Andrew Sharff, Clemens Vonrhein, and Gérard Bricogne

Refinement and rebuilding of pdb entry 1det

To provide as good a target LSSR model as possible to re-solve RNAse T1-pGp based on 5rnt data, PDB entry 1det (Ishikawa et al., 1996) was re-refined and rebuilt. 1det crystallized in the same *I23* space group as 5rnt but with a cell dimension of 88.89 Å compared to 86.47 Å. Ishikawa et al. (1996) solved 1det by molecular replacement using 5rnt as a search model. The original refinement (Ishikawa et al., 1996) used both *X-PLOR* (Brunger, 1992b) and *PROLSQ* (Hendrickson and Konnert, 1981). 1det has a guanosine-2'-phosphate (2'GMP) nucleotide bound and the RNAse T1 is covalently modified by carboxylmethylation of the active site Glu58. The carboxylmethylation blocks the active site phosphate binding site and so alters the 2'GMP binding mode compared to the "canonical" RNAse T1-2'GMP structure 1rnt (Arni et al., 1987).

The model and structure factors for 1det were obtained from the PDB (Berman et al., 2000). Re-refinement used the program *BUSTER* together with rebuilding using the *COOT* program (Emsley et al., 2010). Although 1det has a nominal data resolution of 1.8 Å the *BUSTER* reciprocal space correlation coefficient plot showed poor data quality above 1.95 Å resolution and below 13.5 Å, so these limits were used in refinement. The CCP4 (1994) program *CAD* was used to assign 5% free reflections for R_{free} validation (Brunger, 1992a). It should be noted that the free set was only used for the re-refinement and rebuild rather than throughout structure determination. Following *BUSTER* recommendations for data resolutions better than 2.0 Å, hydrogen atoms were added to both the protein and ligand using the *Reduce* program (Word et al., 1999). A single TLS body for all atoms was used together with individual isotropic atomic B factors. Standard *BUSTER* restraints and default weighting schemes were used. These include Engh and Huber EH99 restraints on amino acid bond lengths and bond angles together with restraints coupling individual temperature factors for bonded atoms. Restraint dictionaries for the carboxymethylated glutamic acid and the 2'GMP ligand were produced using the *grade* program (Smart et al., 2011) based on data obtained from the CSD database using the *Mogul* (Bruno et al., 2004) program. Following refinement the *COOT* program was used to interactively rebuild the model by adjusting some of the side chain rotamers and removing many water molecules. Table S1 shows how the refinement and rebuild lowers the R_{work} by 4% and significantly improves the *MolProbity* (Chen et al., 2010) validation scores.

Refinement and rebuilding shows that in the original 1det pdb structure the protein was in general well modeled, so only small changes were necessary. In addition the two sodium ions placed at crystal contacts in the 1det structure have good density and binding geometries.

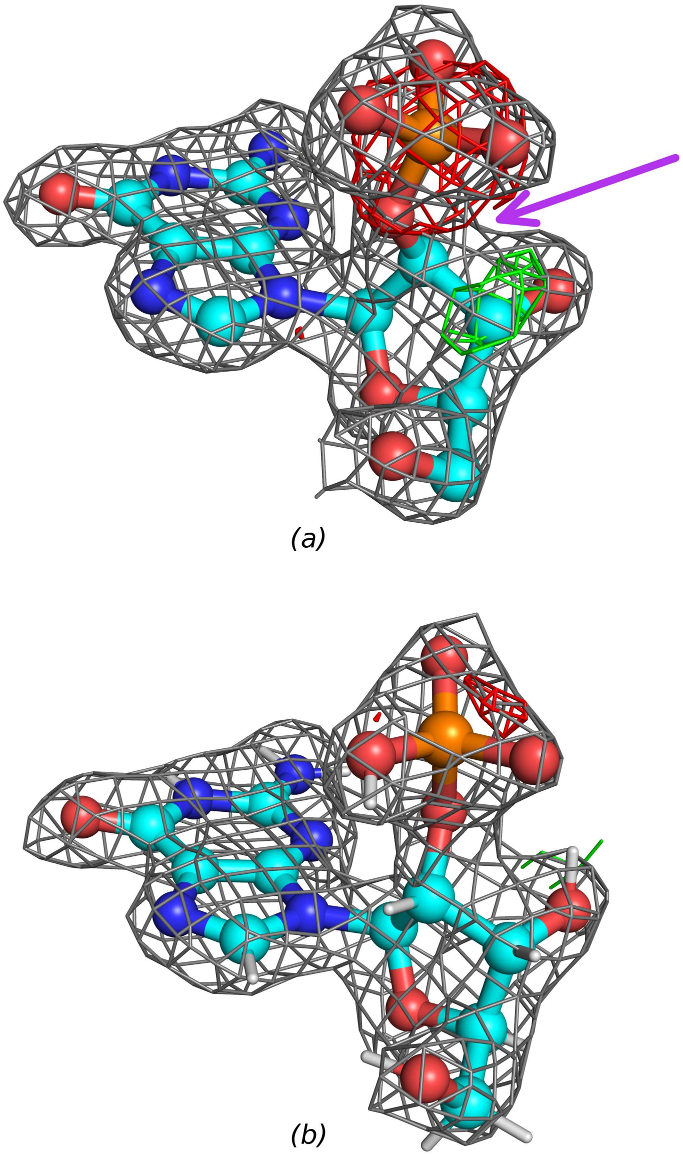

(a)

(b)

Figure S1: **The 2'GMP ligand in 1det and the *BUSTER* density around it.** *(a)* is with the original 1det pdb model, the purple arrow marks the chiral inverted atom C2'. *(b)* shows the better stereochemistry and fit to density for the rebuilt model. 2Fo-Fc density is shown in grey and is contoured at 1.2 sigma. Fo-Fc difference density is contoured at +3.0 sigma in green and -3.0 sigma in red. For clarity the surrounding protein and solvent is not drawn. The figure was produced using *PyMOL* (DeLano, 2009).

Table S1: **re-refinement and rebuilding 1det.**

	1det pdb	rebuilt model
BUSTER R_{work}	0.178	0.138
BUSTER R_{free}	N/A	0.165
Number of water molecules modeled	79	47
MolProbity overall score (Å)	2.66	0.50
MolProbity clash score	16.69	0.00
MolProbity bad rotamers	4/84	0/84
MolProbity Ramachandran outliers	0/101	0/100
MolProbity Ramachandran favored region	96/101	99/100
rms bond length deviation (Å)	0.024	0.010
rms bond angle deviation (degrees)	3.1	1.1
MolProbity residues with bad bonds	1/101	0/100
MolProbity residues with bad angles	7/101	0/100

The overall binding pose of the 2'GMP ligand in the original 1det pdb structure is reasonable with good positioning of the guanine ring, the ribose ring and the phosphate group (Figure S1). However the stereochemistry of the 2'GMP is poor, in that there is a chiral inversion at the 2' carbon atom of the ribose. This inversion is clear if the ribose ring is compared to the 2'GMP ideal coordinates from the ligand expo site (Feng et al., 2004) or the 2'GMP from the "canonical" RNAse structure 1rnt (Arni et al., 1987). *BUSTER* refinement with a *grade* dictionary for 2'GMP fixes the inversion problem. In the initial *BUSTER* refinement negative difference density persisted on the phosphate group in 2'GMP ligand. This could be due to partial ligand occupancy, although alternative explanations are radiation damage or limited disorder of the phosphate. A single group occupancy variable for the 2'GMP ligand was added in the final refinement. The 2'GMP occupancy refines to 0.89 and difference density around the phosphate group is markedly reduced (Figure S1). The real space correlation coefficient for the 2'GMP is increased in the rebuild (Table S2). The ring pucker for the 2'GMP ribose ring provides a useful validation measure because nucleosides have well characterized pucker preferences in small molecule structures (Sun et al., 2004). The ribose ring in the original 1det structure is in the C1'-*exo* conformation that is rarely found in small structures (Sun et al., 2004). Refinement switches the pucker parameters (Table S2) to a favoured C2'-*endo* conformation (Sun et al., 2004). The *Mogul* strangeness score for the ring (Bruno et al., 2004) provides another indication of the ribose ring changing from an unusual to a common conformation.

The rebuilt 1det model has been deposited to the PDB and has been assigned PDB code 3SYU.

Table S2: **1det re-refinement, 2'GMP ligand statistics**

	1det pdb	rebuilt model
BUSTER real space correlation coefficient	0.948	0.962
number of chiral inversions	1	0
ribose ring pseudorotation phase angle P (degrees)[A]	111	154
ribose ring puckering amplitude ν_{max} (degrees)[A]	22	40
ribose ring pucker[A]	C1'-*exo*	C2'-*endo*
ribose *Mogul* ring strangeness score (degrees)	14.2	0.5

[A] found using the PROSIT server *http://cactus.nci.nih.gov/prosit/* (Sun et al., 2004)

Comparison of the rebuilt-5rnt with conformational data from Lenz et al. (1993)

The rebuilt-5rnt model can be compared to conformational data quoted by Lenz et al. (1993) for the same complex solved at 1.8 Å resolution but never deposited to the PDB. Table S3 shows that the conformation of the pGp ligand is similar with torsion and pucker angles with 10 degrees. An exception is for the torsion angles involving the 5' phosphate tail, where larger differences are found. It can be noted that density is weak for the C5' atom (see Fig. 6). The C3'-*endo* ring pucker found is in the center of the well-favored region found in small molecule nucleic acid structures (Sun et al., 2004).

Table S3: **pGp ligand conformation**

	Lenz et al. (1993) Table 2	rebuilt 5rnt-model
P-O5'-C5-C4' torsion angle (degs)	165	-132
O5'-C5'-C4'-C3' torsion angle (degs)	36	62
C5'-C4'-C3'-O3' torsion angle (degs)	93	80
C4'-C3'-O3'-P1 torsion angle (degs)	-150	-156
C5'-C4'-C3'-C2' torsion angle (degs)	-153	-163
C4'-C3'-C2'-O2' torsion angle (degs)	-77	-78
O4'-C1'-N9-C4 torsion angle (degs)	-165	-163
glycosyl bond orientation	*anti*	*anti*
ribose ring pseudorotation phase angle (degs)	13	23
ribose ring pucker	C3'-*endo*	C3'-*endo*

Further comparison is made in Table S4 to ligand contact distances quoted by Lenz et al. (1993) for the pGp and phosphate anion found in the active site. Although the individual contact distances differ by up to 0.5 Å the same pairs of atoms are found in all cases. Solvent molecules are the exception because Lenz et al. (1993) describe 8 water molecule close to pGp and the phosphate. In contrast only one water molecule or small anion "107 unk" was distinguishable with the low resolution data (Fig 6.), this is equivalent to water 133 in Lenz et al. (1993).

Table S4: **ligand contacts distances in Å**

		Lenz et al. (1993) Table 3	rebuilt 5rnt-model
pGp 105 N1	Glu 46 OE1	2.7	2.9
pGp 105 N2	Glu 46 OE2	3.1	2.9
pGp 105 N2	Asn 98 OE2	3.5	3.5
pGp 105 N2	107 unk	3.2	3.5
pGp 105 N2	107 unk	3.0	3.5
pGp 105 O6	Asn 44 N	2.7	2.7
pGp 105 O6	Asn 45 N	2.8	2.9
pGp 105 N7	Asn 43 N	2.9	3.1
pGp 105 N7	Asn 43 ND2	3.3	3.8
pGp 105 O4'	His 40 NE2	3.1	3.3
pGp 105 O4'	His 40 NE2	3.1	3.3
pGp 105 O2P	Lys 41 O	2.6	3.1
pGp 105 O3P	Asn 43 O	3.0	3.7
PO4 106 O	Glu 58 OE1	2.6	2.8
PO4 106 O	Glu 58 OE2	3.0	3.4
PO4 106 O	His 92 NE2	3.3	3.6
PO4 106 O	Arg 77 NE	2.8	3.1
PO4 106 O	Arg 77 NH2	3.0	3.0
PO4 106 O	107 unk	3.1	2.7

References

R. Arni, U. Heinemann, M. Maslowska, R. Tokuoka, and W. Saenger. Restrained least-squares refinement of the crystal structure of the ribonuclease T_1*2'-guanylic acid complex at 1·9 Å resolution. *Acta Crystallographica Section B*, 43(6):548–554, Dec 1987. doi: 10.1107/S0108768187097337.

H. Berman, J. Westbrook, Z. Feng, G. Gilliland, T. Bhat, H. Weissig, I. Shindyalov, and P. Bourne. The Protein Data Bank. *Nucleic Acids Research*, 28(1):235–242, 2000. doi: 10.1093/nar/28.1.235.

A. T. Brunger. Free R value: a novel statistical quantity for assessing the accuracy of crystal structures. *Nature*, 355:472–475, 1992a. doi: 10.1038/355472a0.

A. T. Brunger. *X-PLOR: Version 3.1: A System for X-ray Crystallography and NMR*. Yale Univ Pr, 1992b.

I. Bruno, J. Cole, M. Kessler, J. Luo, W. Motherwell, L. Purkis, B. Smith, R. Taylor, R. Cooper, S. Harris, et al. Retrieval of crystallographically-derived molecular geometry information. *Journal of Chemical Information and Computer Sciences*, 44(6):2133–2144, 2004. doi: 10.1021/ci049780b.

CCP4. The *CCP*4 suite: programs for protein crystallography. *Acta Crystallographica Section D*, 50(5):760–763, Sep 1994. doi: 10.1107/S0907444994003112.

V. B. Chen, W. B. Arendall, III, J. J. Headd, D. A. Keedy, R. M. Immormino, G. J. Kapral, L. W. Murray, J. S. Richardson, and D. C. Richardson. *MolProbity*: all-atom structure validation for macromolecular crystallography. *Acta Crystallographica Section D*, 66(1):12–21, Jan 2010. doi: 10.1107/S0907444909042073.

W. L. DeLano. The PyMOL Molecular Graphics System, version 1.2r1. http://www.pymol.org/, 2009.

P. Emsley, B. Lohkamp, W. G. Scott, and K. Cowtan. Features and development of *Coot*. *Acta Crystallographica Section D*, 66(4):486–501, Apr 2010. doi: 10.1107/S0907444910007493.

Z. Feng, L. Chen, H. Maddula, O. Akcan, R. Oughtred, H. Berman, and J. Westbrook. Ligand Depot: a data warehouse for ligands bound to macromolecules. *Bioinformatics*, 20(13):2153, 2004. doi: 10.1093/bioinformatics/bth214.

W. Hendrickson and J. Konnert. Stereochemically restrained crystallographic least-squares refinement of macromolecular structures. In R. Srinivasan, editor, *Biomolecular Structure, Conformation, Function, and Evolution*, pages 43–57. Pergamon, Oxford, 1981.

K. Ishikawa, E. Suzuki, M. Tanokura, and K. Takahashi. Crystal structure of ribonuclease T1 carboxymethylated at Glu58 in complex with 2'-GMP. *Biochemistry*, 35(25):8329–8334, 1996. doi: 10.1021/bi960493d.

A. Lenz, H.-W. Choe, J. Granzin, U. Heinemann, and W. Saenger. Three-dimensional structure of the ternary complex between ribonuclease t1, guanosine 3,5-bisphosphate and inorganic phosphate at 0.19 nm resolution. *European Journal of Biochemistry*, 211 (1-2):311–316, 1993. ISSN 1432-1033. doi: 10.1111/j.1432-1033.1993.tb19900.x.

O. S. Smart, T. O. Womack, A. Sharff, C. Flensburg, P. Keller, W. Paciorek, C. Vonrhein, and G. Bricogne. grade, version 1.1.1. http://www.globalphasing.com, 2011.

G. Sun, J. Voigt, I. Filippov, V. Marquez, and M. Nicklaus. PROSIT: pseudo-rotational online service and interactive tool, applied to a conformational survey of nucleosides and nucleotides. *Journal of Chemical Information and Computer Sciences*, 44(5):1752–1762, 2004. doi: 10.1021/ci049881+.

J. Word, S. Lovell, T. LaBean, H. Taylor, M. Zalis, B. Presley, J. Richardson, and D. Richardson. Visualizing and quantifying molecular goodness-of-fit: small-probe contact dots with explicit hydrogen atoms1. *Journal of Molecular Biology*, 285(4): 1711–1733, 1999. doi: 10.1006/jmbi.1998.2400.
